# Hesperidin, A Popular Antioxidant Inhibits Melanogenesis via Erk1/2 Mediated MITF Degradation

**DOI:** 10.3390/ijms160818384

**Published:** 2015-08-07

**Authors:** Heun Joo Lee, Woo Jin Lee, Sung Eun Chang, Ga-Young Lee

**Affiliations:** 1 Department of Dermatology, Kangbuk Samsung Hospital, Sungkyunkwan University School of Medicine, Pyeong-dong, Jongno-gu, Seoul 110-746, Korea; E-Mail: heunjoolee111@gmail.com; 2 Department of Dermatology, Asan Medical Center, University of Ulsan College of Medicine, 388-1 Pungnap-dong Songpa-gu, Seoul 138-736, Korea; E-Mail: uucm79@hanmail.net

**Keywords:** hesperidin, anti-melanogenesis, microphthalmia-associated transcription factor (MITF), extracellular signal-regulated kinase 1/2 (Erk1/2), antioxidant

## Abstract

Regulation of melanogenesis has been the focus of treatment for hyperpigmentary skin disorders. Although hesperidin is one of the most well-known, naturally occurring flavonoids with antioxidant and anti-inflammatory effect, its anti-melanogenic effect is not known. The present study aims to determine the anti-melanogenic effect of hespiridin as well as its underlying molecular mechanisms. Melanin contents were measured in normal human melanocytes and B16F10 melanoma cells. Protein and mRNA levels of tyrosinase, microphthalmia-associated transcription factor (MITF), tyrosinase related protein-1 (TRP-1) and TRP-2 were determined. Melanogenesis-regulating signals were examined. In results, hesperidin strongly inhibited melanin synthesis and tyrosinase activity. Hesperidin decreased tyrosinase, TRP-1, and TRP-2 protein expression but increased phospho-extracellular signal-regulated kinase 1/2 (p-Erk1/2) expression. Specific inhibitor of Erk1/2 or proteasome inhibitor reversed the inhibition of melanogenesis induced by hesperidin. Taken together, hesperidin, a popular antioxidant, stimulated Erk1/2 phosphorylation which subsequently degraded MITF which resulted in suppression of melanogenic enzymes and melanin synthesis.

## 1. Introduction

Melanin, the pigment produced by melanocytes, forms the color of human skin and hair. Melanin is transferred to keratinocytes to protect nucleus of keratinocyte against DNA damage by ultraviolet (UV) radiation [1]. Although melanin has an important protective role, overproduction and accumulation of melanin could cause hyperpigmentary disorders such as melasma, solar lentigo, and post-inflammatory hyperpigmentation. Thus regulation of melanogenesis has been the focus of treatment for hyperpigmentary skin disorders and exploration of skin whitening agents [1,2].

Melanogenesis is a complex process with different stages involving various enzymes [2]. Tyrosinase, the key enzyme of melanogenesis, is involved in rate-limiting step for melanin synthesis. Transcription of tyrosinase or tyrosinase related protein-1 (TRP-1) is activated by microphthalmia-associated transcription factor (MITF), a central transcription factor for melanogenesis [3]. MITF is regulated through various signal pathways such as cAMP mediated pathway, MEK/extracellular signal-regulated kinase 1/2 (Erk1/2) pathway, phosphatidylinositol 3 kinase (PI3K)/Akt pathway, and Wnt signaling pathway at transcriptional or post-translational level [3].

Citrus plant extracts worldwide are utilized in cosmetic field, and function primarily as topical skin lightening agents [4]. Previous reports have suggested that citrus plant extracts rich of hesperidin inhibit melanogenesis [5]. It has been reported that hesperidin inhibits melanosome transport by blocking the Rab27A-melanophilin [6]. Although hesperidin, one of the most popular flavonoids has been well studied in all other research fields, its anti-melanogenic effect is not yet demonstrated. Therefore, the objective of this study was to determine the anti-melanogenic effect of hesperidin and its underlying molecular mechanisms.

## 2. Results

### 2.1. Effect of Hesperidin on Melanin Production, Tyrosinase Activity, and Antioxidant Effect

**Figure 1:**
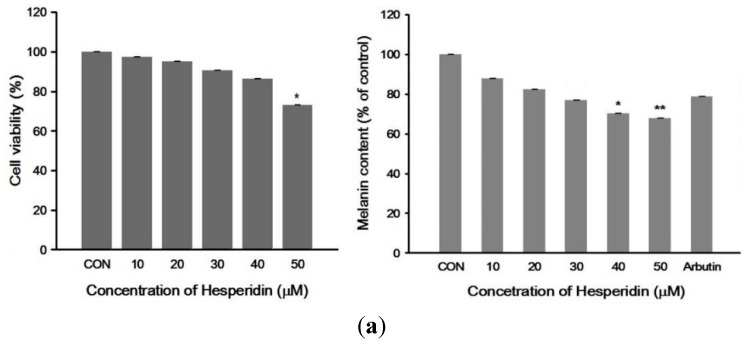

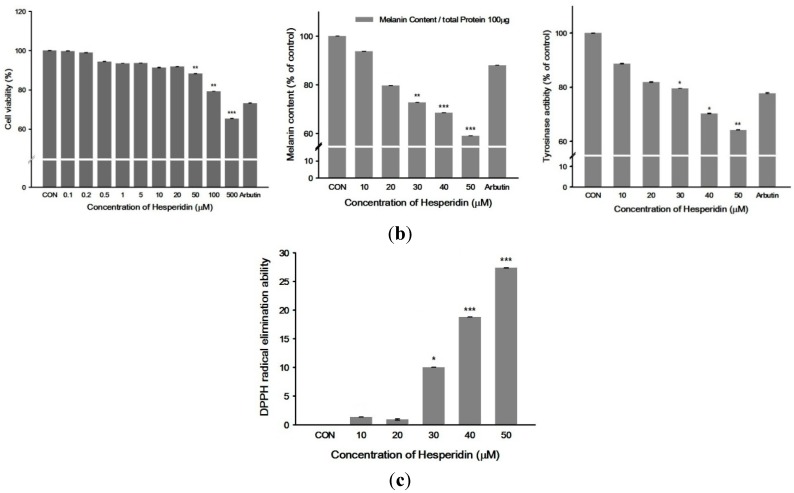
(**a**) Effect of hesperidin on normal human melanocyte viability and cellular melanin contents. Cells were exposed to hesperidin (0–50 μM) and arbutin 50 mg/mL (positive control) for 72 h. Cell viability was determined by 3-(4,5-dimethylthiazol-2-yl)-2,5-diphenyltetrazolium bromide (MTT) assay. Each percentage value in the treated cells was calculated with respect to that in the control cells; (**b**) Effects of hesperidin on B16F10 cell viability, cellular melanin contents and tyrosinase activity. Cells were treated with various concentrations of hesperidin for 48 h. Cell viability was determined by MTT assay. Each percentage value in the treated cells was calculated with respect to that in the control cells (CON); (**c**) DPPH radical scavenging activity of different hesperidin concentrations. Data were expressed as mean ± SD of three independent experiments carried out in triplicates. * *p* < 0.05; ** *p* < 0.01; *** *p* < 0.001 as compared with controls.

Treatment with hesperidin at concentration ranging from 0.1–40 μM had no cytotoxic effect on normal human melanocytes (Figure 1a) and B16F10 cells (Figure 1b). Melanin contents were significantly reduced after exposure to hesperidin in a dose-dependent manner (Figure 1a,b). Likewise, tyrosinase activity was dose-dependently decreased after hesperidin treatment (Figure 1b).

Antioxidant activity of hesperidin was determined in terms of scavenging DPPH, a stable free radical source (Figure 1c). Hesperidin showed potent radical scavenging activity (IC_50_ = 10.60 μg/mL). The IC_50_ of vitamin C, a positive control, was 3.74 μg/mL.

### 2.2. Effect of Hesperidin on Melanogenic Enzymes and MITF

**Figure 2:**
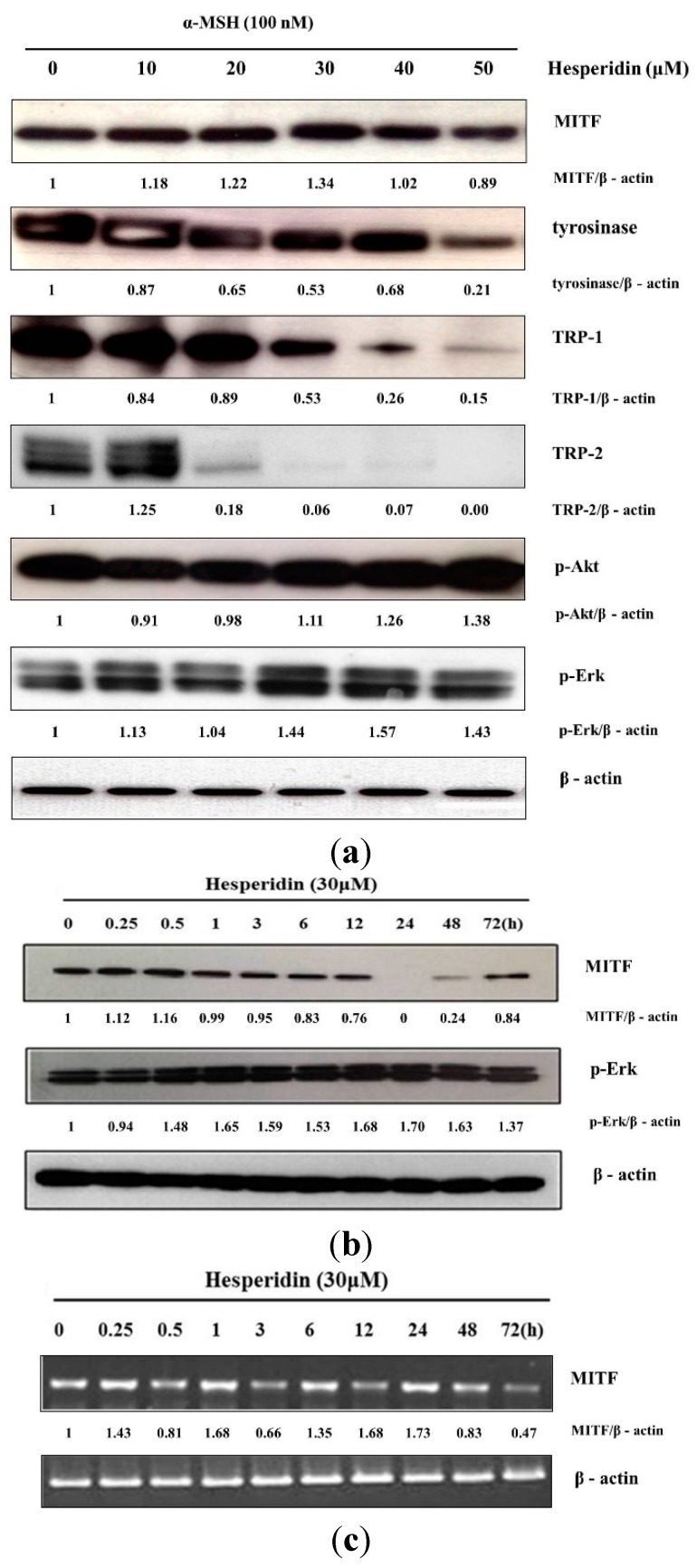
Expression of melanogenesis-related proteins in B16F10 cells treated with hesperidin. The expression levels of MITF, tyrosinase, TRP-1, TRP-2, phospho-Akt and phospho-Erk1/2 proteins were examined by Western blot. (**a**) Cells were treated with hesperidin at different concentrations (0–30 μM) for 72 h and (**b**) treated with 30 μM of hesperidin for the indicated times; (**c**) mRNA levels of MITF was examined by RT-PCR at the indicated times in the presence of 30 μM of hesperidin. Normalization for loading differences was achieved by dividing the densitometry values for individual bands by the densitometry values of β-actin in the same lane.

The expression levels of melanogenesis-related proteins were determined by Western blot in α-melanocyte stimulating hormone (α-MSH) stimulated B16F10 cells. Protein levels of tyrosinase, TRP-1, and TRP-2 were dose-dependently decreased after treatment with hesperidin (Figure 2a). We further investigated the protein level of MITF at different time points in the presence of 30 μM hesperidin. The protein level of MITF was decreased from 12 h and obviously decreased at 24 and 48 h post hesperidin treatment, but it was restored at 72 h (Figure 2b). We further investigated the mRNA level of MITF at different time in the presence of 30 μM hesperidin (Figure 2c). The mRNA level of MITF fluctuated without obvious decrease at 24 or 48 h post hesperidin treatment.

### 2.3. Effect of Hesperidin on Erk1/2 or Akt Signaling Pathways

We found that the inhibitory effect of hesperidin was exhibited through MITF down-regulation. Previous studies reported that the activation of Erk1/2 or Akt induces degradation of MITF, which decreases the level of tyrosinase expression. Therefore, we evaluated whether hesperidin could activate Erk1/2 or Akt signaling pathways. The protein level of p-Erk1/2 was obviously increased after hesperidin treatment while p-Akt was slightly increased (Figure 2a). p-Erk1/2 expression was obviously increased from 30 min after hesperidin treatment and its increase was sustained till 72 h post hesperidin treatment (Figure 2b).

**Figure 3:**
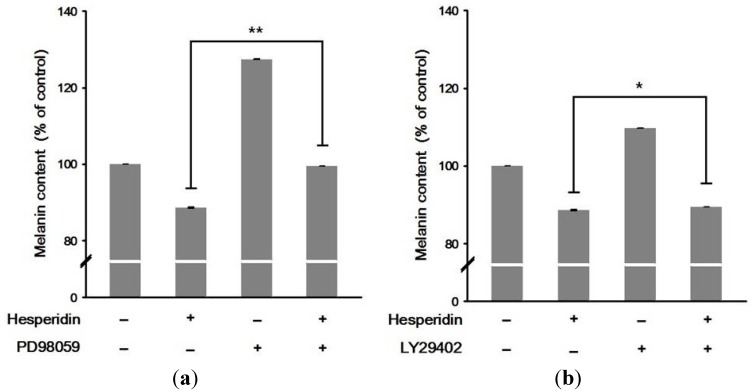
Effect of co-treatment with (**a**) Erk1/2 inhibitor (PD98059) or (**b**) Akt inhibitor (LY29402) in the presence of hesperidin on melanin contents in B16F10 cells. Cells were incubated with Erk1/2 inhibitor PD98059 or Akt inhibitor LY294002 with or without hesperidin. Each percentage value for the treated cells was reported relative to that in α-MSH-stimulated cells (negative control). Data were expressed as mean ± SD of three independent experiments carried out in triplicates. * *p* < 0.05; ** *p* < 0.01 as compared to the one treated with hesperidin (30 μM) only.

**Figure 4:**
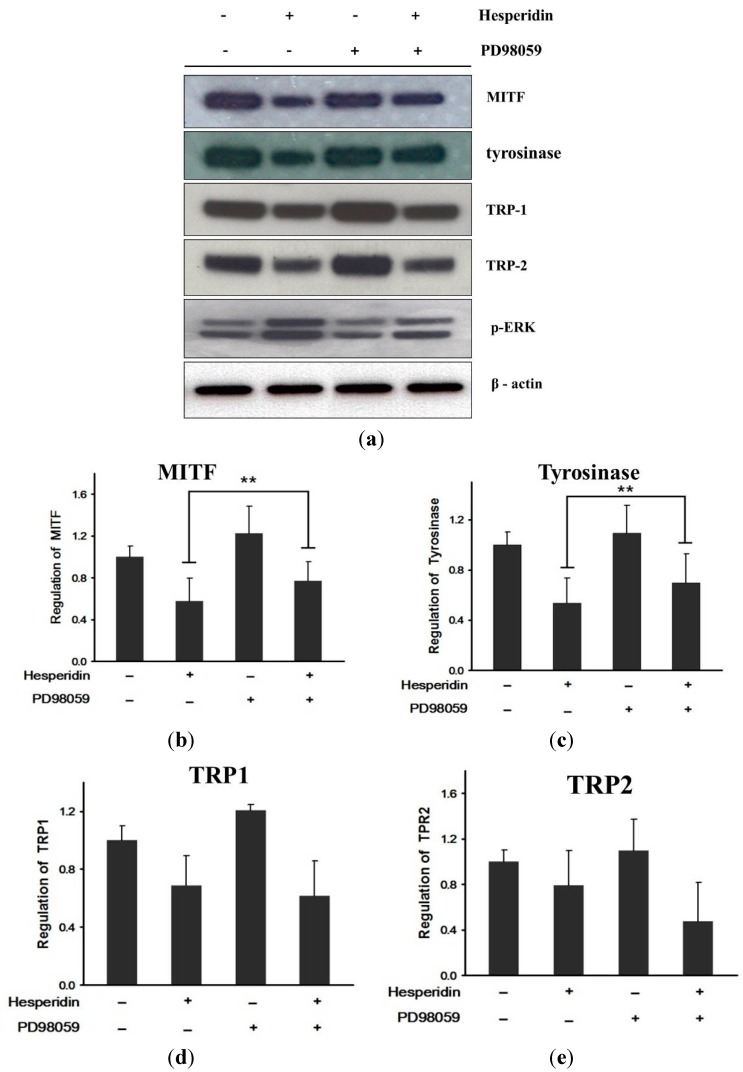
Effect of co-treatment with hesperidin and Erk1/2 inhibitor on melanogenesis-related protein in B16F10 cells. (**a**) Melanogenesis-related protein expressions were analyzed by Western blot; (**b**) MITF; (**c**) Tyrosinase; (**d**) TRP1; (**e**) TRP2 percentage values relative to that in α-MSH-stimulated cells. Data were expressed as mean ± SD of three independent experiments carried out in triplicates. ** *p* < 0.01 as compared to the one treated with hesperidin (30 μM) only.

To confirm whether hesperidin inhibited melanogenesis via the MEK/Erk1/2 and PI3K/Akt pathway, we used PD98059 (Erk1/2 inhibitor) and LY294002 (Akt inhibitor). While Erk1/2 inhibitor significantly abolished the inhibition of melanin synthesis induced by hesperidin treatment, Akt inhibitor did not (Figure 3). Also, the inhibitory effect of hesperidin on MITF and tyrosinase protein expressions was reversed by PD98059 (*p* < 0.01) whereas of the restoration TRP-1 and TRP-2 expression was not seen (Figure 4). These results indicated that hesperidin suppressed melanogenesis through downregulating MITF and downregulation of MITF might be associated with the activation of Erk1/2 signaling pathways.

### 2.4. Effect of Hesperidin on Proteasomal Degradation of MITF

**Figure 5:**
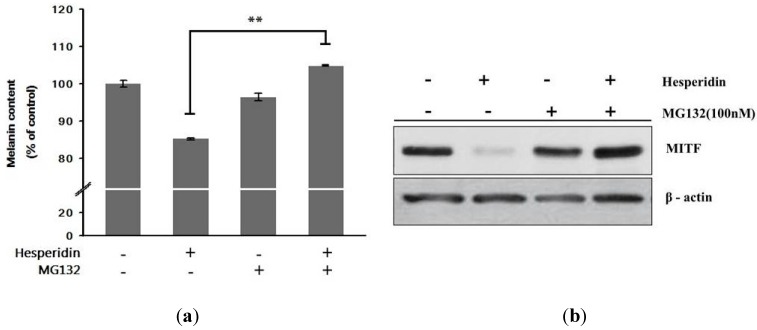
Effect of co-treatment with hesperidin and MG132 (protease inhibitor) on (**a**) melanin contents and (**b**) MITF protein expressions in B16F10 cells. Cells were incubated with MG132 with or without hesperidin. Melanogenesis-related protein expressions were analyzed by Western blot. Each percentage value for the treated cells was reported relative to that in α-MSH-stimulated cells. Data were expressed as mean ± SD of three independent experiments carried out in triplicates. ** *p* < 0.01 as compared to the one treated with hesperidin (30 μM) only.

Then, we examined whether the hesperidin-mediated decrease in MITF protein levels results from its proteasome-dependent degradation of MITF protein. B16F10 cells were incubated with hesperidin for 72 h in the absence or presence of the proteasome-specific inhibitor MG132. Intriguingly, MG132 reversed the suppression of melanin synthesis by hesperidin (Figure 5a) and also prevented the hesperidin-mediated decrease in levels of MITF protein (Figure 5b).

Taken together, these results clearly indicate that hesperidin may inhibit melanin synthesis by upregulating the proteasome-dependent degradation of MITF protein via sustained activation of Erk1/2 signaling pathways.

## 3. Methods

### 3.1. Chemicals and Antibodies

**Figure 6:**
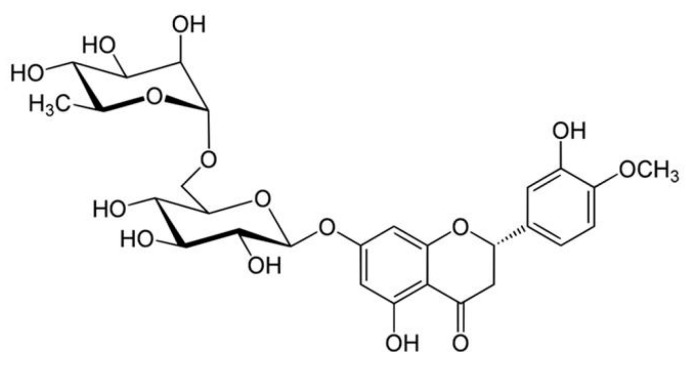
Molecular structure of Hesperidin.

Hesperidin (Figure 6), α-MSH, MTT and L-dihydroxyphenylalanine (L-DOPA) were purchased from Sigma-Aldrich Chemicals Co. (St. Louis, MO, USA). Antibodies recognizing β-actin, phospho-Erk1/2 (p-Erk1/2) and phospho-Akt (p-Akt) were obtained from Cell Signaling Technology (Danvers, MA, USA). MITF, tyrosinase, TRP-1, and TRP-2 antibodies were purchased from Abcam Biotechnology (Abcam, Cambridge, UK). PD98059 (a specific inhibitor of Erk1/2) and LY294002 (a specific inhibitor of PI3K) were purchased from Sigma-Aldrich Chemicals Co. (St. Louis, MO, USA).

### 3.2. Cell Culture, Cell Viability, and Measurement of Antioxidant Effect

Murine B16/F10 melanoma cells (CRL 6475) were obtained from the American Type Culture Collection (Manassas, VA, USA). Cells were cultured in Dulbecco’s modified Eagle’s medium (DMEM) supplemented with 10% fetal bovine serum (FBS) and 1% penicillin/streptomycin at 37 °C in a humidified 5% CO_2_-containing atmosphere. Human epidermal melanocytes (neonatal—moderately pigmented) were cultured in medium 254 supplemented with human melanocyte growth supplement (HMGS; Cascade Biologics, Invitrogen, Carlsbad, CA, USA). Melanocytes at passages between three and seven were used. Melanocytes were fed twice weekly and incubated in a humidified atmosphere at 37 °C and 5% CO_2_.

Cell viability assays were performed using the MTT assay kit according to the manufacturer’s protocol (Dojindo Laboratory, Kumamoto, Japan). Absorbance was measured at 450 nm using a microplate reader (Model 680, Bio-Rad Laboratories, CA, USA).

Antioxidant capacity was evaluated using 1,1-diphenyl-2-picrylhydrazil (DPPH) assay (SIGMA, St. Louis, MO, USA). Radical scavenging capacity of hesperidin was determined according to the method of previous study [7]. Each hesperidin sample stock solution was diluted to a final concentration of 10, 20, 30, 40 and 50 μM. Absorbance 515 nm were measured and converted into percentage antioxidant activity which disappeared with reduction by antioxidant compound. The IC_50_ values were calculated by linear regression of plots, where the abscissa represented the concentration of hesperidin or vitamin C (positive control). Average percent of scavenging capacity was calculated from three replicates.

### 3.3. Measurement of Melanin Contents and Cellular Tyrosinase Activity Assay

After media removal, cells were washed, with cold PBS and lysed with 1 N NaOH in 10% DMSO at 90 °C for 30 min or until fully dissolved. The relative melanin content was measured using a microplate reader at 415 nm.

Cellular tyrosinase activity was estimated by measuring the rate of dopachrome formation of L-DOPA. B6F10 cells grown in 6-well plates were treated with 100 nM α-MSH in the presence of hesperidin for 72 h in DMEM. Tyrosinase substrate, L-DOPA (10 mM), was added to the supernatant sample and tyrosinase. The mixture was incubated at 37 °C for 10 min and the reaction was monitored at 475 nm. The value of each measurement was expressed as a percentage change of the control.

### 3.4. Total RNA Extraction and Reverse Transcription-Polymerase Chain Reaction (RT-PCR)

Isolation of total cellular RNA was performed by using TRIZOL reagent (Invitrogen, Carlsbad, CA, USA). To verify that each primer only hybridized to the target sequence, RT-PCRs were performed using a T-Professional Standard 96 Thermocycler (Biometra GmbH, Goettingen, Germany). PCR products were electrophoresed on 3.0% agarose gel with 1× TAE buffer. Band intensities were quantified using GeneTools V3.07.g software (Syngene, Frederick, MD, USA). All data were normalized to β-actin as a loading control.

### 3.5. Western Blot Analysis

B16F10 cells were cultured in 6-well plates with hesperidin for 72 h with 100 nM α-MSH. Western blot was performed after proteins were separated by 4%–20% gradient sodium dodecyl sulphate-polyacrylamide gel electrophoresis (SDS-PAGE) and transferred to PVDF membranes. Antibodies used for the detection of MITF, tyrosinase, TRP-1, TRP-2, p-Erk1/2, and p-Akt were exposed to appropriate primary antibodies and secondary antibodies. β-Actin was used as a loading control and visualized using enhanced chemiluminescence system (Amersham Biosciences, Piscataway, NJ, USA). Band intensities were quantified using GeneTools.V3.07.g software.

### 3.6. Statistical Analysis

Data are representative of three or more independent experiments replicates and expressed as means ± standard deviation (SD). Each set of data was analyzed using *t*-test followed by Student’s test. The PASW statistics ver.18.0 (SPSS Inc., Chicago, IL, USA) was used for statistical analysis. Statistical significance was considered when *p*-value was less than 0.05.

## 4. Discussion

Hesperidin is a flavonoid antioxidant with diverse biological activities. Flavonoids are naturally occurring low molecular weight polyphenolic compounds [8]. The free hydroxyl on the flavonoid nucleus donates electron to the radicals. Thus, the diverse activities of flavonoids are primarily associated with their antioxidant property. Hesperidin, a major flavonoid in sweet orange and lemon has antioxidant activity and radical scavenging properties [8]. It has been found to reduce superoxide in electron transfer plus concerted proton transfer reaction *in vitro* [8]. Hesperidin has been reported to have the effect on antimicrobial activity [9], anticarcinogenic activity [10], UV protection [11], and radioprotection [12]. Hesperidin is used as an active ingredient of various cosmetic products, including eye cream, anti-redness, anti-wrinkle, and whitening agents [13]. Although over a thousand studies have been published about the bioactivities of hesperidin, few studies have reported the effect of hesperidin on melanogenesis. It is well known that antioxidants have antimelanogenic activity through scavenging reactive oxygen species (ROS) and interacting with melanogenic intermediates [14,15]. Therefore, we investigated the antimelanogenic effect of hesperidin and its underlying mechanism.

In this study, we confirmed the inhibitory effect of hesperidin on melanogenesis. We found that hesperidin decreased tyrosinase activity leading to melanin decrease in a dose-dependent manner. Expression of MITF and MITF’s downstream melanogenesis-related proteins (tyrosinase, TRP-1, and TRP-2) were all decreased in a dose dependent manner after treatment with hesperidin.

As for the central master regulator, MITF is the most important transcription factor for melanogenesis.

Hesperidin clearly decreased MITF protein but the mRNA level of MITF was not consistently decreased in our experiment. Therefore, we presumed that the downregulation of MITF protein may be resulted from post-translational modification.

It is known that MITF is targeted for degradation after its phosphorylation by Erk1/2 or Akt [16,17]. In a previous report, suppression of Erk1/2 induces up-regulation of tyrosinase activity, leading to melanogenesis [18]. In contrast, activation of Erk1/2 phosphorylates MITF at serine 73, followed by ubiquitination and proteasome-mediated degradation of MITF [19]. Akt signaling is more related to regulation of transcriptional level of MITF as activation of Akt pathway downregulates MITF transcription through inhibiting glycogen synthase kinase 3β (GSK-3β) or stimulating mammalian target of rapamycin (mTOR) [20,21].

In our results herein, hesperidin increased Erk1/2 and Akt signaling. Co-treatment with hesperidin and Erk1/2 inhibitor significantly reversed hesperidin-induced decrease of MITF and tyrosinase protein expression, and finally, melanin contents. However, co-treatment with hesperidin and Akt inihibitor did not show the significant reverse of hesperidin-induced decrease of melanin contents.

Furthermore, MITF is targeted for proteasomal degradation [16,17] and a proteasome inhibitor, MG-132 was reported to prevent downregulation of MITF, suggesting a role of proteasome in antimelanogenic effect [22]. In our experiment, MG-132 reversed the hesperidin-induced decrease of MITF protein expression, and resulting melanin contents. Thus, these results suggest that hesperidin inhibited melanogenesis through the acceleration of Erk1/2-mediated MITF proteasomal degradation.

A recent report showed that hesperidin could block melanosome transport but did not inhibited melanin synthesis or tyrosinase activity in their experiment condition [6]. However, in our experiment, for the first time, we clearly demonstrated that hesperidin significantly reduced melanogenesis in normal human melanocytes as well as in B16F10 cells. Hesperidin could be a promising, safe and healthy skin whitening agent with more benefit with simultaneous antioxidant and skin barrier strengthening effects.
